# Hypertension diagnosis and management in Bamako, Mali

**DOI:** 10.2471/BLT.23.290658

**Published:** 2025-02-27

**Authors:** Issiaka Camara, Mohamed Traore, Mamoudou Maiga, Dike Ojji, Jane Holl, Souleymane Coulibaly, Mark D Huffman

**Affiliations:** a School of Medicine and Dentistry, University of Sciences Technics and Technology of Bamako, Bamako, 10000, Mali.; b Feinberg School of Medicine, Northwestern University, Chicago, United States of America (USA).; c Department of Internal Medicine, Cardiovascular Research Unit, University of Abuja, Abuja, Nigeria.; d Department of Neurology, University of Chicago, Chicago, USA.; e Department of Cardiology, Point G University Teaching Hospital, Bamako, Mali.; f Cardiovascular Division, Washington University School of Medicine, St Louis, USA.

## Abstract

**Problem:**

The capacity and site readiness for delivering hypertension management services in Mali were unknown, hindering the effective implementation of the World Health Organization (WHO) HEARTS technical package for cardiovascular disease management.

**Approach:**

We selected one tertiary and two secondary hospitals to be assessed. From December 2021 to January 2022, hospital cardiologists collected data on indicators of capacity and site readiness using an adapted version of the WHO service availability and readiness assessment questionnaire. The study team verified the collected data through site inspection and review of administrative documents.

**Local setting:**

Mali, a low-income country with a population of 22 395 489, had an estimated hypertension prevalence among adults of 35% in 2019. Most people with hypertension receive care from primary care clinicians, but there are no national hypertension treatment guidelines.

**Relevant changes:**

The tertiary hospital had a larger workforce (392 personnel) compared to the two other sites (124 and  182 personnel, respectively) and treated approximately three times more patients with high blood pressure (324 patients versus 106 and 132 patients, respectively). Diuretics and centrally acting agents were the only antihypertensive medications available at all three sites. While all three sites had the capacity to diagnose and confirm hypertension, only one site was fully equipped to provide comprehensive hypertension treatment.

**Lessons learnt:**

Political engagement is important for expanding service availability and readiness assessments across health-care facilities, and supporting the implementation and funding of the HEARTS package. Improving access to antihypertensive medications will be essential to ensuring better treatment options for patients.

## Résumé

**Problème:**

La capacité et l’état de préparation des sites pour la prise en charge de l’hypertension au Mali étaient inconnus, ce qui entravait la mise en œuvre efficace du guide technique HEARTS de l’Organisation mondiale de la santé (OMS) pour la prise en charge des maladies cardiovasculaires.

**Approche:**

Nous avons sélectionné un hôpital de soins tertiaires et deux hôpitaux de soins secondaires pour procéder à notre évaluation. De décembre 2021 à janvier 2022, les cardiologues de ces hôpitaux ont recueilli des données sur les indicateurs de capacité et de préparation des sites à l’aide d’une version adaptée du questionnaire d’évaluation de la disponibilité et de la capacité opérationnelle des services (SARA) de l’OMS. L’équipe de l’étude a vérifié les données collectées en inspectant les sites et en examinant les documents administratifs.

**Environnement local:**

Le Mali, pays à faible revenu comptant 22 395 489 habitants, avait une prévalence de l’hypertension estimée à 35% chez les adultes en 2019. La plupart des personnes souffrant d’hypertension sont prises en charge par des cliniciens de soins primaires, mais il n’existe pas de directives nationales sur le traitement de l’hypertension.

**Changements significatifs:**

L’hôpital de soins tertiaires disposait d’un effectif plus important (392 personnes) que les deux autres sites (124 et 182 personnes, respectivement) et traitait environ trois fois plus de patients souffrant d’hypertension artérielle (324 patients contre 106 et 132 patients, respectivement). Les diurétiques et les agents à action centrale étaient les seuls antihypertenseurs disponibles dans les trois sites. Si ceux-ci avaient la capacité de diagnostiquer et de confirmer l’hypertension, seul un site était entièrement équipé pour fournir un traitement complet de l’hypertension.

**Leçons tirées:**

L’engagement politique est important pour étendre les évaluations de la disponibilité des services et de l’état de préparation dans les établissements de soins de santé, ainsi que pour soutenir la mise en œuvre et le financement de l’ensemble des mesures du guide technique HEARTS. L’amélioration de l’accès aux antihypertenseurs sera essentielle pour garantir de meilleures options thérapeutiques aux patients.

## Resumen

**Situación:**

Se desconocía la capacidad y el nivel de preparación de los establecimientos de salud en Malí para ofrecer servicios de manejo de la hipertensión, lo que dificultaba la implementación efectiva del paquete técnico HEARTS de la Organización Mundial de la Salud (OMS) para el manejo de las enfermedades cardiovasculares.

**Enfoque:**

Se seleccionaron un hospital terciario y dos hospitales secundarios para su evaluación. Entre diciembre de 2021 y enero de 2022, cardiólogos hospitalarios recopilaron datos sobre indicadores de capacidad y preparación de los establecimientos mediante una versión adaptada del cuestionario de evaluación de disponibilidad y preparación de los servicios de la OMS. El equipo del estudio verificó los datos recopilados mediante inspección *in situ* y revisión de documentos administrativos.

**Marco regional:**

Malí, un país de bajos ingresos con una población de 22 395 489 habitantes, tenía en 2019 una prevalencia estimada de hipertensión del 35% entre los adultos. La mayoría de las personas con hipertensión reciben atención de médicos de atención primaria, pero no existen guías nacionales de tratamiento para la hipertensión.

**Cambios importantes:**

El hospital terciario contaba con una mayor dotación de personal (392 profesionales) en comparación con los otros dos establecimientos (124 y 182, respectivamente) y atendía aproximadamente tres veces más pacientes con hipertensión arterial (324 pacientes frente a 106 y 132, respectivamente). Los únicos medicamentos antihipertensivos disponibles en los tres establecimientos eran diuréticos y agentes de acción central. Aunque los tres centros tenían la capacidad para diagnosticar y confirmar la hipertensión, solo uno de ellos estaba completamente equipado para proporcionar un tratamiento integral.

**Lecciones aprendidas:**

El compromiso político es fundamental para ampliar las evaluaciones de disponibilidad y preparación de los servicios en los establecimientos de salud y para apoyar la implementación y financiación del paquete HEARTS. Mejorar el acceso a los medicamentos antihipertensivos será esencial para garantizar mejores opciones de tratamiento para los pacientes.

## ملخص

المشكلة كانت القدرة وجاهزية الموقع لتقديم خدمات علاج ارتفاع ضغط الدم في مالي غير معروفين، مما أعاق التنفيذ الفعال لحزمة HEARTS الفنية التابعة لمنظمة الصحة العالمية (WHO) لعلاج أمراض القلب والأوعية الدموية.

الأسلوب لقد اخترنا مستشفى ثالثيًا واثنين من المستشفيات الثانوية للتقييم. من ديسمبر/كانون أول 2021 إلى يناير/كانون ثاني 2022، قام أطباء القلب بالمستشفيات بجمع بيانات حول مؤشرات القدرة وجاهزية الموقع، باستخدام نسخة معدلة من استبيان تقييم توفر الخدمة والجاهزية التابع لمنظمة الصحة العالمية. وتحقق فريق الدراسة من البيانات التي تم جمعها من خلال فحص الموقع ومراجعة الوثائق الإدارية.

المواقع المحلية مالي هي دولة منخفضة الدخل يبلغ عدد سكانها 22395489 نسمة، وكان لديها معدل انتشار لارتفاع ضغط الدم بين البالغين بنسبة %35 في عام 2019. يتلقى معظم المصابين بارتفاع ضغط الدم الرعاية من أطباء الرعاية الأولية، ولكن لا توجد إرشادات وطنية لعلاج ارتفاع ضغط الدم.

التغيّرات ذات الصلة كان لدى المستشفى الثالثي قوى عاملة أكبر (392 موظفًا) مقارنة بالموقعين الآخرين (124 و182 موظفًا على التوالي)، وعالج ما يقرب من ثلاثة أضعاف عدد المرضى الذين يعانون من ارتفاع ضغط الدم (324 مريضًا مقابل 106 و132 مريضًا على التوالي). كانت مدرات البول والعوامل ذات التأثير المركزي هي الأدوية الخافضة للضغط الوحيدة المتوفرة في جميع المواقع الثلاثة. وفي حين أن جميع المواقع الثلاثة لديها القدرة على تشخيص وتأكيد ارتفاع ضغط الدم، إلا أن موقعًا واحدًا فقط كان مجهزًا بالكامل لتقديم علاج شامل لارتفاع ضغط الدم.

الدروس المستفادة المشاركة السياسية مهمة لتوسيع نطاق توفر الخدمة وتقييم الجاهزية عبر مرافق الرعاية الصحية، ودعم تنفيذ وتمويل حزمة HEARTS. سيكون تحسين فرصة الحصول على الأدوية الخافضة لضغط الدم ضروريًا لضمان خيارات علاج أفضل للمرضى.

## 摘要

**问题:**

马里提供高血压管理服务的能力以及医疗机构准备情况尚不明确，这不利于世界卫生组织 (WHO) 用于心血管疾病管理的“健康促进行动：高血压和心血管疾病风险综合管理” (HEARTS)技术包的有效实施。

**方法:**

我们选取了一家三级医院和两家二级医院来进行评估。在 2021 年 12 月至 2022 年 1 月期间，医院心脏病专家使用《WHO 服务可用性和准备情况评估问卷（修订版）》收集了有关能力和医疗机构准备情况指标的数据。通过现场检查和审查管理文件，研究小组核实了所收集的数据。

**当地状况:**

马里是一个低收入国家，总人口为 22,395,489 人，2019 年的成人高血压患病率约为 35%。大多数高血压患者由初 级保健机构的临床医生提供护理服务，但马里并无国家高血压治疗指南。

**相关变化:**

该三级医院的工作人员数量（392 人）比其他两个站点（分别为 124 人和 182 人）都要多，且其治疗的高血压患者人数也比其他两个站点多三倍左右（324 名患者分别对比 106 名和 132 名患者）。利尿剂和中枢性作用药物是从这三个站点均可购得的唯一抗高血压药物。虽然这三个站点都有能力诊断和确诊高血压，但只有一个站点充分配备了可提供高血压综合治疗服务的设备。

**经验教训:**

政治参是确保在医疗卫生机构扩大服务可用性和准备情况评估范围、支持推行心血管疾病管理技术包并为此提供资金的重要前提条件。提高抗高血压药物的可及性对于确保患者获取更好的治疗方案至关重要。

## Резюме

**Проблема:**

Отсутствие данных о вместимости и степени готовности медицинских объектов для осуществления лечения гипертензии в Мали препятствовало эффективному внедрению разработанного Всемирной организацией здравоохранения (ВОЗ) технического пакета HEARTS, предназначенного для лечения сердечно-сосудистых заболеваний.

**Подход:**

Для оценки были выбраны одна больница третичного звена и две больницы вторичного. С декабря 2021 года по январь 2022 года кардиологи в этих больницах собирали данные об индикаторах вместимости, пропускной способности и готовности медицинских объектов, используя адаптированный вариант анкеты ВОЗ для оценки доступности услуг и готовности к их оказанию. Исследовательская группа проверила собранные данные путем инспекции объектов и изучения административных документов.

**Местные условия:**

Мали, страна с низким уровнем доходов и населением в 22 395 489 человек, имела по состоянию на 2019 год частоту гипертензии среди взрослых на уровне 35%. Большинство пациентов с гипертензией лечились у своих участковых врачей, но национальные рекомендации по лечению гипертензии отсутствовали.

**Осуществленные перемены:**

Больница третичного звена имела больше персонала (392 человека) по сравнению с двумя другими (124 и 182 сотрудника соответственно) и приняла за этот период почти втрое больше пациентов с высоким давлением (324 пациента против 106 и 132 соответственно). Во всех трех больницах для лечения гипертензии были доступны только диуретики и препараты центрального действия. Хотя все три больницы были в состоянии диагностировать и подтвердить гипертензию, только одна была полностью оборудована для всеобъемлющего лечения этого состояния.

**Выводы:**

Политическая заинтересованность – важный фактор расширения возможности оценивания медицинских учреждений по параметрам доступности медицинского обслуживания и готовности к оказанию таких услуг, а также поддержки внедрения и финансирования закупок пакетов HEARTS. Улучшение доступа к антигипертензивным препаратам – существенный фактор гарантии улучшения возможностей лечения пациентов.

## Introduction

In Mali, a low-income country in sub-Saharan Africa, noncommunicable diseases have become a considerable public health concern. In 2019, the estimated age-standardized prevalence of hypertension was 35% among adults aged 35–79 years. Despite the high prevalence of hypertension, 47% of individuals with hypertension were undiagnosed, and only 36% of those diagnosed received treatment and 15% had controlled their hypertension.^1^ These detection and treatment failures contribute to increased risks of stroke, heart disease and renal failure among people with hypertension.^1^

To guide governments, health workers and policy-makers to implement effective strategies for cardiovascular disease management in primary care settings, the World Health Organization (WHO) has developed the HEARTS technical package, which has been successfully implemented in over 30 countries.^2^^,^^3^ The package contains six modules: (i) healthy-lifestyle counselling; (ii) evidence-based protocols; (iii) access to essential medicines and technology; (iv) risk-based cardiovascular disease management; (v) team-based care; and (vi) systems for monitoring. To prepare for implementation of the package, health ministries need to conduct capacity and site readiness assessments of cardiovascular disease management in their respective countries. However, no such assessment has been conducted in Mali. Here we describe how we evaluated the capacity and readiness in Mali to implement the HEARTS package.

## Local setting

Mali has a population of 22 395 489, with 4 227 569 residents living in the capital Bamako.^4^ Mali’s structured health-care system consists of primary care, which refers patients to secondary care, with tertiary care as the final referral level. The health ministry develops strategies and roadmaps, makes operational decisions and mobilizes resources from public and financial partners.^5^ In 1987, the health ministry adopted the Bamako Initiative, aiming to provide affordable medicines and health services to the public.^6^

Most patients with hypertension in Mali receive care from primary care clinicians, especially in rural areas.^7^ However, health workers, including physicians and cardiologists, are scarce and primarily work in urban hospitals. The primary strategic plan for addressing noncommunicable diseases focuses on strengthening health workers’ capacity to prevent and treat hypertension and diabetes in health-care facilities. As part of this effort, noncommunicable disease technical officers from different regional health offices trained 1292 health workers from 28 secondary health-care districts, marking an important step towards implementing the HEARTS package in the country.^8^ However, there are no national hypertension treatment guidelines in Mali. 

## Approach

To evaluate the capacity and readiness of hospitals in Bamako to diagnose and manage people with hypertension, the cardiology department of the Point G University Hospital in Bamako collaborated with Northwestern University, United States of America (USA), Washington University in St Louis (USA), the University of Chicago (USA) and the University of Abuja (Nigeria). We used the adapted WHO service availability and readiness assessment questionnaire from the hypertension treatment programme in Nigeria.^9^ The questionnaire is designed to evaluate and monitor the capacity, readiness and availability of health-care facilities, including key indicators such as the availability of basic equipment, essential medicines and diagnostic capacity.^10^ While originally intended for primary health-care centres, we modified the questionnaire to include tertiary centres to ensure that well-equipped centres in Mali are also prepared for HEARTS implementation. We chose this questionnaire because it was built upon other tools, such as the Kaiser Permanente implementation package and WHO’s Service Availability Mapping, and the questionnaire leverages best practices and insights gained from numerous countries that have conducted health facility assessments, including in sub-Saharan Africa.^1^ Additionally, we had previous experience using the questionnaire in similar health-care settings, demonstrating its feasibility.^9^

Using an open-source web tool, we randomly chose three hospitals out of seven hospitals in Bamako. Two were secondary regional referral hospitals and one was a tertiary hospital, Point G University Hospital. Between December 2021 and January 2022, one cardiologist at each referral hospital and two cardiologists at the tertiary hospital used the adapted service availability and readiness assessment questionnaire to manually collect data from medical and administrative records, and recorded the information on paper. Subsequently they entered the data in the software Research Electronic Data Capture (Vanderbilt University, Nashville, USA). We later synchronized the data to the online version of the software, hosted securely by the University of Bamako. The cardiologists were remunerated for their work. 

Because paper-based patient records are prone to inaccuracies, we conducted a comparison of patient charts from the cardiology department with outpatient data at the hospital's first point of entry. This approach enabled the collection of accurate patient, visit-level data. Additionally, we conducted site visits to assess the availability of medications, materials and information systems, identifying gaps and opportunities for improvement in ascertainment and clinical care.

We obtained ethical approval from the University of Bamako.

The direct and indirect costs for this assessment were 4000 United States dollars (US$).

## Relevant changes

We found high availability of trained personnel. All three sites had staff members trained to diagnose hypertension and provide treatment. Hypertension treatment algorithms were present at two sites (Table 1). The tertiary hospital had a larger workforce (392 personnel, including specialist physicians, nurses, pharmacists and paramedics) compared to the two secondary hospitals (124 and 182 personnel, respectively; Table 2). In the three months before data collection, the tertiary hospital treated 324 patients with high blood pressure – three times more than the secondary hospitals (106 and 132 patients, respectively). However, the tertiary hospital had fewer newly registered patients compared to the other two sites in the month preceding data collection.

**Table 1:** Capacity and site readiness to implement a hypertension control programme in three hospitals in Bamako, Mali, December 2021–January 2022

Characteristic	No.
**Availability of personnel and training**	
General practitioners	3
Specialist doctors	3
Nonphysician clinicians and paramedics	3
Nurses	3
Pharmacists	3
Licensed personnel to provide hypertension care	3
Blood pressure monitor	3
**Information systems**	
Functional telephone line	0
Access to email or internet	0
Functional computer	3
Electronic patients files	0
**Availability of blood pressure lowering medications**	
Angiotensin converting enzyme inhibitor	1
Angiotensin receptor blocker	0
β blocker	0
Calcium channel blockers	2
Central acting agent	3
Fixed dose combinations	0
Diuretic	3
Vasodilator	0
30-day treatment regimens	1
**Hypertension service delivery**	
Screening for high blood pressure	3
Diagnosing high blood pressure	3
Confirming high blood pressure	3
Dispensing initial medication for hypertension	1
Dispensing follow-up medication for hypertension	1
Monitoring patients with hypertension	3
Providing long-term care for patients with hypertension	3
**Equipment and supplies for hypertension**	
Guidelines	0
Treatment algorithms	2
Information, education and communication materials	1
Functional blood pressure monitor	3

Note: Non-physician clinicians include midwives, state-registered nurses and physician assistants.

**Table 2:** Overall site readiness for hypertension screening and management in three hospitals in Bamako, Mali, December 2021–January 2022

Characteristic	No (%)			
	Point G Hospital	Referral health centre I	Referral health centre II	Total
**Personnel**				
General practitioners	16 (32)	19 (39)	14 (29)	49 (100)
Specialist doctors	149 (79)	22 (12)	17 (9)	188(100)
Nurses	119 (42)	64 (22)	101 (36)	284 (100)
Nonphysicians clinicians and paramedics	103 (60)	19 (11)	49 (29)	171 (100)
Pharmacists	5 (72)	1 (14)	1 (14)	7 (100)
Total	392 (100)	124 (100)	182 (100)	698 (100)
**Facility outpatient visits past month**				
Total visits	5105 (15)	15 567 (46)	13 162 (39)	33 834 (100)
Total visits for hypertension	26(3)	217 (29)	519 (68)	762 (100)
**Hypertension service delivery and materials**				
Patients screened for hypertension, past 3 months	688 (45)	440 (30)	390 (25)	1 518 (100)
Patients diagnosed with hypertension, past 3 months	125 (26)	172 (36)	186 (38)	483 (100)
Patients treated for hypertension, past 3 months	324 (58)	106 (19)	132 (23)	562 (100)
Functioning blood pressure monitor	48 (52)	30 (32)	15 (16)	93 (100)

Note: Non-physician clinicians include midwives, state-registered nurses and physician assistants.

While all three sites had the capacity to diagnose, confirm and treat hypertension, only the tertiary hospital provided information, education and communication materials to patients (Table 1).

None of the sites had an electronic health record system, telephone or internet access, leading to the manual documentation of clinical data. However, functional sphygmomanometers and computers were present at all sites.

Diuretics and central acting agents were the only hypertension medications present at all three sites, but only one site had a 30-day supply available (Table 1). Calcium channel blockers and central acting agents were the only two medication classes with sufficient stock for a single 30-day treatment, also available at only one centre. Other essential antihypertensive medications listed in the *WHO Model list of* *essential medicines*, including angiotensin-converting enzyme inhibitors, angiotensin receptor blockers, β blockers or two-drug fixed-dose combination therapy, were not available at any of the three sites.^11^

Detailed information on the availability of personnel, equipment, information systems, medications, and outpatient visit frequency is available in the online repository.^12^

## Lessons learnt

In addition to providing general health-care services, all three sites had an adequately trained health workforce capable of screening, diagnosing and managing hypertension and cardiovascular disease. However, due to medication shortages, only one site was fully equipped to treat hypertension. While all sites had some blood pressure medications available, they were unable to consistently dispense a 30-day supply, hindering their capacity to provide comprehensive hypertension care. 

Because the selected sites, a tertiary and two referral centres, are among the best-equipped in Bamako, the results may not be generalizable to the broader health system in Mali, particularly primary care facilities, where service capacity and readiness are likely lower. Challenges, such as limited access to essential antihypertensive medications, the absence of standardized treatment guidelines and the lack of electronic health records to support care delivery and coordination, may hinder an effective implementation of HEARTS in the Malian health system. Box 1 summarizes the main lessons learnt from the assessment to inform HEARTS implementation.

Box 1 **Summary of main lessons learnt**

- Political engagement is crucial to expand service availability and readiness assessments and prepare health-care facilities for implementation and funding of the HEARTS package.
- Including antihypertensive medications in a revised Bamako Initiative^6^ could improve the accessibility of these medications.
- Enhanced coordination of care will be needed to implement WHO treatment guidelines^13^ through a national hypertension treatment protocol.

To further guide HEARTS implementation, the project team plans to evaluate the availability of essential antihypertensive medications in district and community health centres in Bamako. These findings, along with insights into system coordination challenges, will provide researchers and policy-makers with valuable data to inform strategies for effective implementation.

Political engagement is essential for expanding service availability and readiness assessments, as well as for ensuring successful implementation in health-care facilities and sustainable funding of the HEARTS package. Ongoing collaboration between the health and higher education ministries has already facilitated the initiation of cardiologist training programmes in Mali. However, the Bamako Initiative requires revisions to strengthen governance and oversight of the supply chain, increase subsidies for essential medicines and foster partnership with international donors and the private sector to co-finance medicine distribution programmes.

The health ministry should prioritize noncommunicable disease management by expanding service availability and readiness assessments across more health-care facilities, as these are necessary steps for the successful implementation of the HEARTS package in Mali. Addressing key barriers, such as fragmented care coordination, medication shortages, the absence of a national hypertension treatment protocol and reliance on paper-based records, is essential to achieving effective implementation.
